# The presence of a systemic inflammatory response predicts poorer survival in patients receiving adjuvant 5-FU chemotherapy following potentially curative resection for colorectal cancer

**DOI:** 10.1038/sj.bjc.6603185

**Published:** 2006-05-23

**Authors:** J E M Crozier, R F McKee, C S McArdle, W J Angerson, J H Anderson, P G Horgan, D C McMillan

**Affiliations:** 1 University Department of Surgery, Royal Infirmary, Glasgow G31 2ER, UK

**Keywords:** C-reactive protein, albumin, Dukes stage, 5-FU adjuvant chemotherapy, survival, colorectal cancer

## Abstract

There is increasing evidence that the presence of a systemic inflammatory response plays an important role in survival following curative resection for colorectal cancer. The present study evaluated the relationship between C-reactive protein concentrations and survival in a cohort of patients receiving adjuvant 5-fluorouracil (5-FU) chemotherapy following potentially curative resection for colorectal cancer. In all, 222 patients undergoing potentially curative resection for colorectal cancer were studied. Of these, 50 patients received adjuvant 5-FU-based chemotherapy. Circulating concentrations of C-reactive protein were measured prior to surgery. The minimum follow-up was 15 months; the median follow-up of the survivors was 38 months. During this period 61 patients died, 32 patients of their cancer and 29 of intercurrent disease. In those patients who did not receive adjuvant chemotherapy, age (*P*<0.001), Dukes stage (*P*<0.05) and an elevated C-reactive protein (*P*<0.01) were significantly associated with survival. In those patients who did receive adjuvant chemotherapy, an elevated C-reactive protein concentration (*P*<0.01) was significantly associated with survival. The presence of a systemic inflammatory response is an independent predictor of poor outcome in patients receiving adjuvant 5-FU-based chemotherapy following potentially curative resection for colorectal cancer.

Colorectal cancer remains the second commonest cause of cancer death in Western Europe and North America. Overall survival is poor; even in those patients who undergo potentially curative resection, more than one-third die within 5 years (McArdle and Hole, 2002). In view of these poor results there is increasing interest in the use of adjuvant chemotherapy.

Conventionally, in patients with primary operable colorectal cancer, the decision whether or not to offer adjuvant 5-fluorouracil (5-FU)-based chemotherapy is primarily based on the patient's age, pathological stage and fitness to tolerate chemotherapy. However, even in this selected cohort, the impact of chemotherapy on outcome is unpredictable. Therefore, there is continuing interest in prognostic factors that better reflect clinical outcome (Cascinu *et al*, 2003; Benson *et al*, 2004).

It has been demonstrated that the presence of a systemic inflammatory response, as evidenced by elevated circulating concentrations of C-reactive protein, is associated with increased recurrence and poor survival, independent of Dukes stage, in patients undergoing potentially curative surgery for colorectal cancer (McMillan *et al*, 1995, 2003; Nielsen *et al*, 2000). However, in these studies, few patients had received adjuvant chemotherapy.

It is therefore of considerable interest to examine whether this poor outcome might also be found in patients receiving adjuvant chemotherapy. Indeed, an elevated C-reactive protein has recently been shown to be associated with poorer survival in patients receiving chemotherapy for advanced lung cancer (Forrest *et al*, 2004) and renal cancer patients (Bromwich *et al*, 2004).

Therefore, the aim of the present study was to evaluate the relationship between the systemic inflammatory response and survival in a prospective cohort of patients receiving adjuvant 5-FU chemotherapy following potentially curative resection for colorectal cancer.

## PATIENTS AND METHODS

### Patients

Patients with histologically proven colorectal cancer who, on the basis of laparotomy findings and preoperative abdominal computed tomography, were considered to have undergone a potentially curative resection between January 1999 and June 2004 at Glasgow Royal Infirmary were included in the study. The tumours were staged using conventional Dukes classification (Dukes and Bussey, 1958). Patients who had preoperative radiotherapy were excluded from the study since radiotherapy has been reported to evoke a systemic inflammatory response (Cengiz *et al*, 2001; Koc *et al*, 2003).

Patients were selected for 5-FU-based chemotherapy following discussion in the multidisciplinary group and taking into account tumour pathology, comorbidity and also patients' wishes. This was predominantly administered using the Mayo regimen for six cycles (O'Connell *et al*, 1997).

A blood sample was taken for routine laboratory measurement of C-reactive protein measurement immediately prior to surgery. The limit of detection of the assay was a C-reactive protein concentration lower than 6 mg l^−1^. The coefficient of variation, over the range of measurement, was <5%, as established by routine quality-control procedures. At this time no patient showed clinical evidence of infection or other inflammatory condition.

The study was approved by the Research Ethics Committee, Royal Infirmary, Glasgow.

### Statistics

Comparisons between groups of patients were carried out using contingency table analysis (*X*^2^) as appropriate. Grouping of the variables age and C-reactive protein was carried out using standard thresholds (O'Gorman *et al*, 2000; Scottish Cancer Intelligence Unit, 2000). Survival analysis of the group variables was performed using the Cox proportional hazard model. Deaths up to 31st August 2005 were included in the analysis. Multivariate survival analysis, including all covariates was performed using a stepwise backward procedure to derive a final model of the variables that had a significant independent relationship with survival. To remove a variable from the model, the corresponding *P*-value had to be >0.10. Analysis was performed using SPSS software (SPSS Inc., Chicago, IL, USA).

## RESULTS

Two hundred and twenty-two patients undergoing potentially curative resection for colorectal cancer were studied (Table 1). The majority of patients were aged 65 years or more, had colonic tumours and had C-reactive protein concentration in the normal range (⩽10 mg l^−1^) prior to surgery.

Of the 222 patients, 50 received adjuvant 5-FU-based chemotherapy (Table 1). Those patients who received chemotherapy were younger (*P*<0.001), more likely to be male (*P*<0.10), were more likely to have Dukes C disease (*P*<0.001) and did not have hypoalbuminaemia (*P*⩽0.01). The groups were similar in terms of site and C-reactive protein concentration.

The minimum follow-up was 15 months; the median follow-up of the survivors was 38 months. During this period 61 patients died, 32 patients of their cancer and 29 of intercurrent disease. On univariate survival analysis, in those patients who did not receive adjuvant chemotherapy, age (*P*<0.001), Dukes stage (*P*<0.05) and an elevated C-reactive protein (*P*<0.01) were significantly associated with survival (Table 2). In those patients who did receive adjuvant chemotherapy, an elevated C-reactive protein concentration (*P*<0.01) was significantly associated with survival.

On multivariate survival analysis, in those patients who did not receive adjuvant chemotherapy, age (*P*<0.05) and an elevated C-reactive protein (*P*<0.05) were independently associated with survival (Table 2). In those patients who did receive adjuvant chemotherapy, an elevated C-reactive protein concentration (*P*<0.05) was independently associated with survival.

## DISCUSSION

In the present study, an elevated C-reactive protein concentration was associated with poorer survival, independent of age and Dukes stage, in patients receiving adjuvant chemotherapy following potentially curative resection for colorectal cancer. These results would suggest that the systemic inflammatory response, as evidenced by an elevated C-reactive protein concentration, is an important factor in determining outcome in patients receiving adjuvant 5-FU-based chemotherapy.

The basis of the relationship between the systemic inflammatory response and poor survival in patients undergoing potentially curative resection for colorectal cancer is not clear. The presence of an elevated C-reactive protein concentration may simply reflect a nonspecific inflammatory response secondary to tumour necrosis or local tissue damage. However, these elevated C-reactive protein concentrations do not appear to resolve following potentially curative surgery in the majority of patients (McMillan *et al*, 2003). Also, an elevated C-reactive protein concentration 3–6 months following curative resection also has independent prognostic value (McMillan *et al*, 1995, 2003). Therefore, these data suggest that the systemic inflammatory response participates in the progression of metastatic disease in patients with colorectal cancer.

There are a number of possible mechanisms by which this could occur. Firstly, that an elevated C-reactive protein identifies those patients with an impaired T-lymphocytic response, since poor infiltration of gastrointestinal tumours appears to be associated with poor outcome (Jass *et al*, 1987; Nielsen *et al*, 1999) and an elevated C-reactive protein concentration has recently been shown to be inversely associated with T-lymphocyte subset infiltration (Canna *et al*, 2005). An alternative explanation is that an elevated C-reactive protein concentration may identify those patients with a proangiogenic environment, since increased angiogenesis is associated with poor outcome in patients with colorectal cancer (Salmon *et al*, 2005) and circulating concentrations of vascular endothelial growth factor are directly associated with C-reactive protein (Xavier *et al*, 2006). Clearly, both these inflammatory mechanisms may be related and promote unrestrained tumour growth and the dissemination required for the greater malignant potential associated with an elevated C-reactive protein concentration.

In the present study, an elevated C-reactive protein concentration also predicted poor outcome in those patients receiving adjuvant 5-FU-based chemotherapy. However, it has long been recognised that progressive weight loss is associated with poor tolerance to chemotherapy. For example, Andreyev *et al* (1998) in a study of over 1500 patients who were to receive chemotherapy for gastrointestinal cancer showed that prior weight loss was an independent prognostic factor, and patients with weight loss received less chemotherapy and developed more dose-limiting toxicity. They concluded that there was a need to conduct nutritional intervention studies in these patients.

More recently, it has been shown that the presence of an ongoing systemic inflammatory response, as evidenced by an elevated C-reactive protein concentration, predicts the progressive nutritional decline of the patient with advanced gastrointestinal cancer (Lundholm *et al*, 1994; McMillan *et al*, 1999; O'Gorman *et al*, 1999). Moreover, recent work has shown that the activity of the enzyme cytochrome *P*450 3A, which is involved in the biotransformation of more than half of all drugs currently available, is compromised in patients with an elevated C-reactive protein concentration (Rivory *et al*, 2002; Slaviero *et al*, 2003; Baker *et al*, 2004). It may therefore be that there is a need to carry out studies to moderate the systemic inflammatory response rather than nutritional intervention in patients receiving chemotherapy.

Irrespective of the mechanisms involved, we believe that the presence or absence of a systemic inflammatory response should be evaluated as a possible influence on outcome in future trials of adjuvant chemotherapy in patients with colorectal cancer and should be used in the stratification of patients. This is however a small study and further larger studies are required to confirm these results.

In summary, the presence of a systemic inflammatory response appears to be an independent predictor of poor outcome in patients receiving adjuvant 5-FU-based chemotherapy following potentially curative resection for colorectal cancer.

## Figures and Tables

**Table 1:** Clinicopathological characteristics in patients undergoing potentially curative surgery with and without adjuvant 5-FU chemotherapy for colorectal cancer

	**No adjuvant 5-FU**	**Adjuvant 5-FU**	***P*-value**
	172 (%)	50 (%)	
*Age group (years)*			
<65	40 (23)	26 (52)	
65–74	58 (34)	18 (36)	
⩾75	74 (43)	6 (12)	<0.001

*Sex*			
Male	88 (51)	33 (66)	
Female	84 (49)	17 (34)	0.064

*Site*			
Colon	100 (58)	28 (56)	
Rectum	72 (42)	22 (44)	0.788

*Dukes stage*			
A	23 (13)	0 (0)	
B	96 (56)	9 (18)	
C	53 (31)	41 (82)	<0.001

*C-reactive protein*			
⩽10 mg l^−1^	95 (55)	32 (64)	
>10 mg l^−1^	77 (45)	18 (36)	0.270

*Albumin*			
⩾35 g l^−1^	132 (77)	45 (90)	
<35 g l^−1^	20 (12)	0 (0)	0.01
Alive	126 (74)	35 (70)	

*Dead*			
Cancer specific	23 (13)	9 (18)	
Intercurrent	23 (13)	6 (12)	0.709

**Table 2:** Clinicopathological characteristics in patients undergoing potentially curative surgery and adjuvant 5-FU chemotherapy for colorectal cancer (*n*=222) and survival

	**No adjuvant chemotherapy (*n*=172)**		**Adjuvant chemotherapy (*n*=50)**	
	**HR (95% CI)**	***P*-value**	**HR (95% CI)**	***P*-value**
*Univariate analysis*				
Age (<65/65–74/⩾75) years	2.33 (1.48–3.68)	<0.001	0.73 (0.31–1.71)	0.464
Sex (male/female)	1.37 (0.77–2.46)	0.287	1.55 (0.55–4.36)	0.411
Site (colon/rectum)	1.23 (0.69–2.21)	0.477	1.19 (0.43–3.29)	0.735
Dukes stage (A/B/C)	1.75 (1.06–2.89)	0.029	3.36 (0.44–25.85)	0.245
C-reactive protein (⩽10, >10 mg l^−1^)	2.39 (1.32–4.34)	0.004	6.68 (2.05–21.72)	0.002
Albumin (⩾35/<35 g l^−1^)	1.42 (0.59–3.40)	0.433		

*Multivariate analysis*				
Age (<65/65–74/⩾75) years	1.87 (1.13–3.09)	0.015	1.21 (0.47–3.15)	0.693
Sex (male/female)	1.08 (0.55–2.09)	0.828	0.92 (0.26–3.22)	0.894
Site (colon/rectum)	1.57 (0.81–3.07)	0.185	1.15 (0.31–4.27)	0.834
Dukes stage (A/B/C)	1.39 (0.82–2.36)	0.219	2.56 (0.31–21.21)	0.384
C-reactive protein (⩽10, >10 mg l^−1^)	2.10 (1.04–4.25)	0.039	5.57 (1.32–23.51)	0.019
Albumin (⩾35/<35 g l^−1^)	1.18 (0.48–2.88)	0.721		
